# Investigating Biomechanical Postural Control Strategies in Healthy Aging Adults and Survivors of Stroke

**DOI:** 10.3390/biomechanics4010010

**Published:** 2024-03-05

**Authors:** Lara A. Thompson, Roni A. Romero Melendez, Ji Chen

**Affiliations:** Center for Biomechanical & Rehabilitation Engineering, Biomedical Engineering Program, School of Engineering and Applied Sciences, University of the District of Columbia, 4200 Connecticut Ave. NW, Washington, DC 20008, USA

**Keywords:** biomechanics, elderly, aging, balance, postural control

## Abstract

As the aging populations, both nationwide and worldwide, rapidly increase, falls leading to unintentional injury and death subsequently increase. Thus, developing an understanding of biomechanical postural control strategies used to maintain balance in aging healthy adults, and those that have suffered stroke, are critical. Here, we were interested in how one’s body segments stabilize relative to one another, and in space, in order to maintain balance. To accomplish this goal, we studied 30 healthy individuals and 8 survivors of stroke between 60 and 85 years old, both before and after several weeks of sensory training. Motion capture data were acquired to assess participants’ body kinematics during walking: forward (easiest), forward-tandem, backward, and backward-tandem walking (most challenging). Deviations (via the observation of the absolute angle with deviations, or AADs) of the head, thorax, and lumbar areas relative to an earth vertical reference, as well as how one body segment stabilized in space or relative to the inferior body segment (via the observation of anchoring indices, or AIs), were explored. The results provide metrics (AADs and AIs) that can assess aging posture. Further, the results show an initial indication that, for aging individuals, training could lead to improved head and body stabilization in space.

## 1. Introduction

Falls are the second leading cause of unintentional injury-related deaths worldwide, and, according to the United States Centers for Disease Control and Prevention, 25% of Americans over 65 years old fall each year [1,2]. By the year 2030, researchers predict that there will be seven deadly falls every hour, with the financial toll expected to increase to over USD 101 billion [3]. For persons 65 years and older, in over half of this population, stroke reduces mobility [4]; the majority of strokes occur in people over 65 years old. Thus, older individuals that are survivors of stroke have an even higher fall risk. For these reasons, there is an imperative need to understand postural control used to maintain balance in aging populations.

Balance refers to an individual’s ability to maintain equilibrium. Postural control involves the act of maintaining, achieving, or restoring a state of balance during any posture or activity. Postural control, sensory and motor responses are tied to maintaining postural orientation and postural equilibrium. It is well known that the sensory information used for the control of posture is mainly from the vestibular, visual, and somatosensory systems, e.g., [5,6]. Aging leads to the degradation of these systems, which thereby affects postural control [7–9]. The visual system utilizes inputs from one’s eyes to obtain information about the surrounding environment. The vestibular system utilizes cues from the inner ear to sense angular and linear head movements for equilibrium. Somatosensation mediates body sensations that are associated with touch, proprioception, and interoception and is composed of the following sub-modalities: mechanoreception (sensing pressure or stretch), thermoception (sensing heat or cold), nociception (sensing pain), and proprioception (sensing body position) [10].

Postural regulation is complex and requires the coordination and control of rotational movements around hundreds of joints by means of several hundreds of muscles [11]. Proprioception is the sense through which we perceive the position and movement of our body, including our sense of equilibrium and balance [12]. Proprioceptive signals from the leg muscles provide the primary source of information for postural control [9]. Proprioception allows one to walk in a coordinated manner, without conscious thought; for example, it also allows one to touch one’s finger to one’s nose when their eyes are closed. Due to a combination of natural age-related changes to the nerves, joints, and muscles, the risk of proprioception loss increases as we age [13]. Decreased proprioception results in a reduction in body positioning in space. Stroke and other neurological issues can affect and cause proprioceptive disorders, which lead to falls, balance issues, and uncoordinated movements. Poor proprioception can also lead to one’s avoidance of certain movements or activities, such as climbing stairs or walking on uneven surfaces, and a fear of falling [14]. Postural strategy is linked to proprioception in that it involves the use of a coordinated set of body movements in order to maintain balance. For quiet, stationary standing, common strategies include an ankle strategy (sway about one’s ankle joint), a hip strategy (counter rotating about the hip joint) and stepping (for larger deviations in one’s center of mass in order to prevent a fall) [15].

There is an interest in studying training tied to aging balance, and only a few example studies are described here. A study by Komiya et al. [16] studied chronic survivors of stroke that were either controls or underwent feedback training on walking ability for 6 weeks. Their performance was measured by the Timed Up and Go (TUG), which included timed walking trials and self-reported, survey questionnaires (modified Gait Efficacy Scale and Falls Efficacy Scale). They found a positive influence on self-confidence and gait performance; however, body kinematics were not measured nor explored. A study by DeLuca et al. [17] in chronic survivors of stroke involved the evaluation of the effectiveness of trunk and balance training using a robotic versus traditional physical therapy. Training was conducted over 15 sessions (3 times per week = 5 weeks). Assessments used were the Berg Balance Scale, Trunk Impairment Scale, Mini-BESTest, Robot-Based Evaluation, static balance test (stationary standing for 30 s, both with eyes open and with eyes closed), a dynamic balance test on an unstable platform, a reactive balance test, a proprioceptive control test (reaching task), and sit to stand (timed sitting and standing trials). However, body kinematics and relative body movements were not measured. A study by Reimann et al. [18] used a different approach from the previously mentioned studies and investigated the interactions between different age-related factors of walking from a neural control perspective. They introduced a model that generated walking movement patterns to simulate empirical studies. Comparisons between the model and empirical results could be used to understand balance control. However, here, we were specifically interested in how aging individuals move one body segment relative to another, and to a global reference frame, while walking both pre- and post-training.

There were limited papers that dealt with aging balance and chronic stroke that, in particular, assessed walking pre- and post-training via angular metrics and kinematics. The most relevant studies are described below. Previous research done by Fabio et al. [14] showed that the stabilization of one’s head in space (minimizing head movements) was thought improve the interpretation of vestibular inputs, especially when visual and somatosensory inputs were distorted or limited. Head stability was examined by comparing peak-to-peak (PP) pitch head motion, but not AI or AADs as we have done here, with the PP displacement of the center of force in the sagittal plane—this was referred to as the head mobility score (HMS). Other body movements were not examined. For the sway-driven tilt of the visual surround, platform eyes-open, and platform eyes-closed, elderly subjects showed a decrease in HMS for the first two conditions and no change for the last condition; however, young subjects showed a decrease, no change, and no change in their HMS for the conditions above, respectively.

To investigate how one body segment moved relative to the other and in space, we examined walking in aged adults, both healthy and those that were survivors of stroke. Implemented postural strategies could involve the choice of the frame of reference for the basis of the equilibrium control (e.g., support surface leading feet to head (or bottom-up recruitment) or the Earth’s gravitational vector from head to feet (or top-down)). Frames of reference in terms of stabilization include geocentric (orientation to Earth vertical), egocentric (reference is defined with respect to some part of the observer—for example, orientation of the head with respect to the body), and exocentric (frame of reference external to the observer) [19]. Here, we were interested specifically in how the older individuals stabilized relative to Earth vertical (via absolute angle with deviations across subjects, AADs) and in terms of body segment positions relative to one another (via anchoring indices, AIs). Anchoring indices (or AIs) are measures that indicate how stable a specific anatomical segment of the human body is with respect to the global coordinate system or to the anatomical segment directly below (inferior to) it (i.e., degree of dependency between two consecutive segmental movements), e.g., [19,20].

Previously, Nadeau et al. [19] investigated the head and trunk equilibrium strategies of healthy, but not aged, adults while walking forward and backward for eyes open/closed visual conditions and hard vs. soft (foam) support surface conditions. The kinematics of the head, spine, and pelvis segments were recorded while walking and anchoring indices (AIs) and absolute angular dispersions were computed. Of note is that the acronym of “AADs” in [19] differs than our definition described here; we were interested in the absolute angle with the deviations across subjects, as opposed to angular dispersion as in [19]. For the healthy adults, the roll AIs of the head and pelvis were positive; this was interpreted to indicate stabilization in space. Further, for backward walking on foam, it was observed that the stability in space increased. Conversely, spinal segments were observed to have negative AIs and thus were interpreted to indicate stabilization on the underlying segment. Further, increasing walking difficulty induced a rigid (‘en bloc’ motion), or an AI approaching 0, of the spinal segments. In older adults, while walking with eyes open or closed, Cromwell et al. [21] investigated the influence of vision on head stabilization. They observed that during eyes-closed walking, the peak head velocity, alongside the walking velocity, decreased. However, head movements relative to body movements were not investigated here, solely focusing on head velocity.

We aimed to investigate head and trunk equilibrium strategies for older individuals that were healthy or were survivors of stroke, pre- and post-sensory training. Our research questions included how the postural strategy differed in healthy older adults and older adults who had suffered a stroke, before and after several weeks of sensorimotor training. For the above, we examined the relative movement of body segments using AADs and segmental AIs. Here, we examined segmental AIs, specifically displaying up and down movements. The walking condition difficulty was modified by having participants walk forward or backward in a regular or tandem stance. We hypothesized that increased head stabilization in space would be the result for the more challenging walking conditions and that there would be observable differences in healthy individuals compared to survivors of stroke. The basis for this hypothesis was that, in a previous study by Nadeau et al. [19]; they had observed that there was head stabilization in space for adults during more challenging walking conditions (i.e., backward walking).

## 2. Materials and Methods

All study activities presented here were conducted within the Center for Biomechanical & Rehabilitation Engineering (CBRE) at the University of the District of Columbia (Institutional Review Board approved protocol #979744–1). Here, we observed the postural strategy, in terms of AADs and AIs, predominantly in healthy older individuals but also survivors of stroke. Participants were recruited via flyer postings around the university campus, as well as by word of mouth. Prior to taking part in the study, all participants gave their informed consent.

Thirty healthy and eight stroke participants (60–85 years old) enrolled in this study; one stroke participant and three healthy participants withdrew from the study. Thus, pre- and post-training results are reported for 27 healthy individuals (69.6 ± 5.5 years old) and 7 survivors of stroke 66.1 ± 8.6 years old). Demographics are detailed in Tables 1 and 2 [22]. Inclusion criteria included a score greater than 25 on the Mini-Mental State Examination (all participants scored > 28). For survivors of stroke, they were at least 6 months post-stroke and able to ambulate without external assistance or a cane, orthosis, or walker for at least 15 m.

### 2.1. Training Protocol

**Figure 1.**
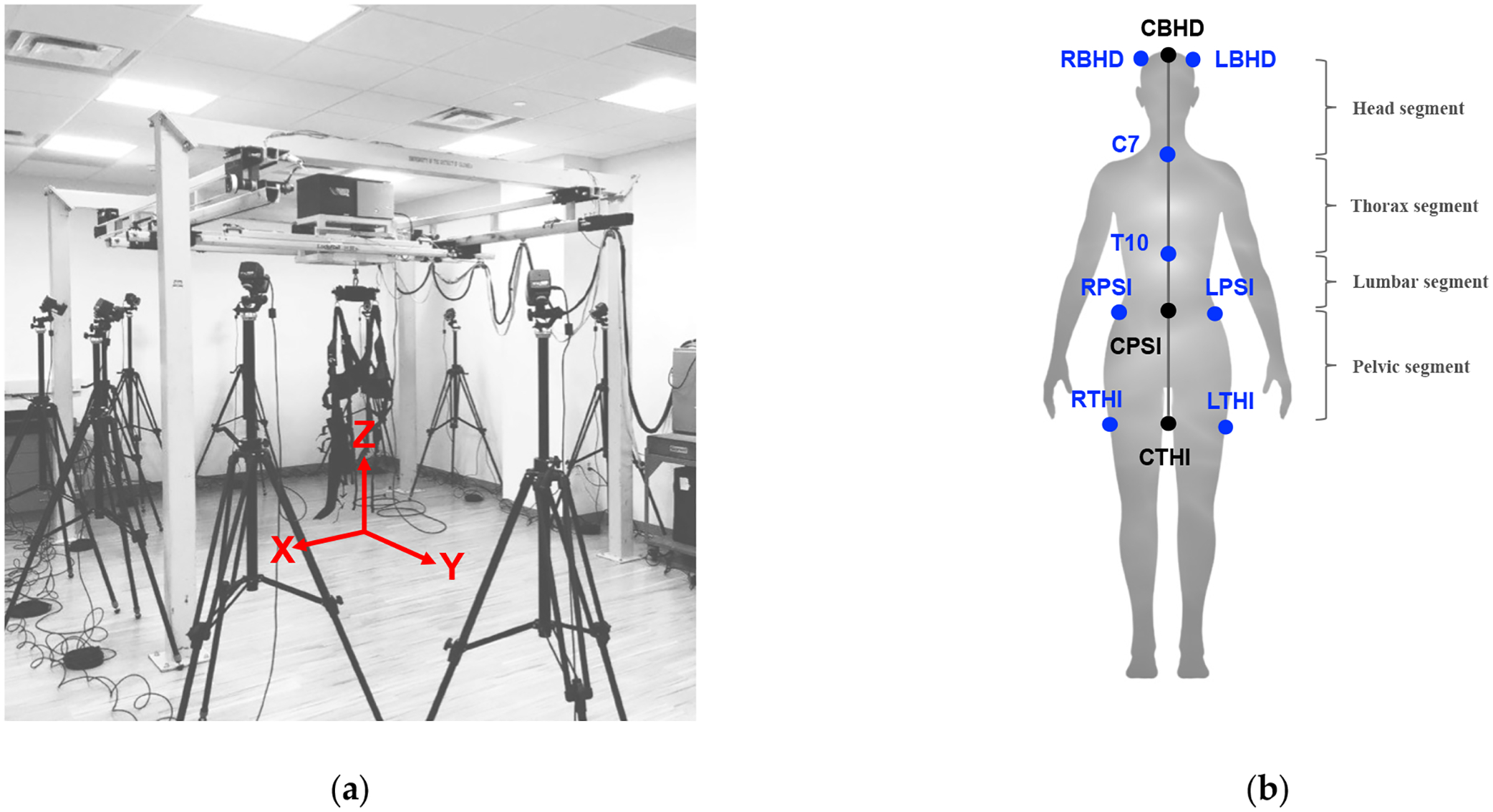
(**a**) Laboratory coordinate system. (**b**) Arrangement of the 8 reflective markers (blue) attached to the participant; black markers shown are computed, virtual landmarks.

Within the CBRE, participants each underwent individual 6-week exercise routines, which consisted of two 30-min sessions/week. It is well known that the visual, somatosensory, and vestibular systems affect balance. Thus, the training modified the vision (eyes closed/eyes open, respectively) and support surface somatosensory cues (hard surface or foam surface). We also included various standing and walking tasks; these tasks increased in difficulty relative to the increase in training weeks. The base-of-support was modified from a double-leg stance (easiest) and tandem stance to single-leg stance (most challenging). The training involved eyes-open/-closed activities for walking exercises (forward and backward for regular and tandem walking, left and right side-stepping), foam exercises (standing, isolated leg exercises, squats, and walking on hard surface) on either dense or thick compliant foam, and walking over obstacles [22]. Participants wore a harness from a NaviGAITor robotic device (shown in Figure 1a) during training to safeguard them from any injury due to a potential fall, and, further, they were spotted by the experimenters for the duration of the training sessions. Training was monitored by the principal investigator as well as the CBRE research associates.

### 2.2. Assessment Protocol

The assessment was conducted before and after training. Participants were asked to perform either forward or backward (regular) walking or tandem (heel-to-toe) walking. Participants performed two regular walking trials and two tandem walking trials, with a 3 m distance per trial for both forward and backward walking. They were asked to walk at their regular walking speed while facing forward. The *y*-axis (Figure 1a) was aligned with the walking direction. Vicon Motion Capture (Oxford, UK) was used to record body kinematic measures (marker displacements), which were acquired and preprocessed using the Vicon Nexus 2.0 software. In order to record body kinematics, each participant was instrumented with reflective markers to match the “plug-in-gait” marker placement template in the Vicon Nexus 2.0 software. Marker trajectories were recorded at 100 Hz from twelve T40 cameras. Marker position information was acquired as referenced to the x, y, and z positions (Figure 1a).

From the Vicon Nexus plug-in gait model, there are eight infrared markers, indicated in blue (Figure 1b), used to study head and trunk stabilization. We were most interested in the medial or centerline movements of the body from the head through the upper thigh. Thus, ‘virtual’ landmarks (center back head (CBHD), center posterior superior iliac (CPSI), and center thigh (CTHI)) were computed by taking the average or center (denoted as “C” in the abbreviations) movements of the right back head (RBHD) and left back head (LBHD), right posterior superior iliac (RPSI) and left posterior superior iliac (LPSI), and right thigh (RTHI) and left thigh (LTHI), respectively (Figure 1b). The landmarks used to study angular movements were CBHD, C7 (location of the 7th cervical vertebra), T10 (location of the 10th thoracic vertebra), CPSI, and CTHI, as shown in Figure 1b. These landmarks were used for the calculation of the angular movements (i.e., AADs and AIs) of four segments: the head segment (CBHD to C7), thoracic segment (C7 to T10), lumbar segment (T10 to CPSI), and pelvic segment (CPSI to CTHI).

The absolute angle with deviation across participants (AAD) was computed according to a global or laboratory coordinate system (x, y, z axes shown in Figure 1a). Of note is that the acronym of “AADs”, for example in [19,20], differs than our definition described here. Our calculated AADs of each segment were based on the absolute positions of two markers that defined the segment and were calculated with respect to three rotational axes: roll (rotation about the *y*-axis), pitch (rotation about the *x*-axis), and yaw (rotation about the *z*-axis), as defined in [20]. For a demonstration of how the head segment AAD was calculated, using the landmarks CBHD and C7, we display the AAD head segment equations below (Equations (1)–(3)). It is important to note that the equations changed depending on the body segment.


(1) $$\theta_{H_{a}}^{R}=tan^{-1}\left(\frac{X_{\text{CBHD}}-X_{C7}}{Z_{\text{CBHD}}-Z_{C7}}\right)$$

(2) $$\theta_{H_{a}}^{P}=tan^{-1}\left(\frac{Y_{\text{CBHD}}-Y_{C7}}{Z_{\text{CBHD}}-Z_{C7}}\right)$$

(3) $$\theta_{H_{a}}^{Y}=tan^{-1}\left(\frac{X_{\text{CBHD}}-X_{C7}}{Y_{\text{CBHD}}-Y_{C7}}\right)$$

In Equation (1), $\theta_{H_{a}}^{R}$ is the head absolute roll. In Equation (2), $\theta_{H_{a}}^{P}$ is the head absolute pitch. In Equation (3), $\theta_{H_{a}}^{Y}$ is the head absolute yaw. In Equations (1)–(3), ***X***, ***Y***, ***or Z***_***CBHD***_ are the the x, y, or z-displacement, respectively, of the CBHD, and ***X***, ***Y***, ***or Z***_***C***7_ are the the x, y, or z-displacement, respectively, of the C7.

AIs were computed and varied between −1 and +1: +AI = better stabilization in space than the lower segment; −AI = better stabilization on the lower segment than in space; 0 AI = en bloc (or rigid) movement between segments. For demonstration, we show the equations for the head segment. The head relative roll angle (of the head relative to the thorax) was calculated using Equation (4).


(4) $$\theta_{r}^{H}=\theta_{H_{a}}^{R}-\theta_{T_{a}}^{R}$$

In Equation (4), $\theta_{r}^{H}$ is the roll of the head relative to the thorax, $\theta_{H_{a}}^{R}$ is the head absolute roll, and $\theta_{T_{a}}^{R}$ is the thorax absolute roll. With the head absolute roll angle (Equation (1)) and the relative angle of the head to the thorax (Equation (4)), the AI was determined for the head segment (Equation (5)) using the standard deviations of the relative roll angular distribution and absolute head roll, followed by normalization. In Equation (5), $\sigma \left(\theta_{r}^{H}\right)$ is the standard deviation of the relative roll angular distribution and $\sigma \left(\theta_{Ha}^{R}\right)$ is the standard deviation of the absolute head roll.


(5) $$AI(H)=\frac{\sigma^{2}\left(\theta_{r}^{H}\right)-\sigma^{2}\left(\theta_{H_{a}}^{R}\right)}{\sigma^{2}\left(\theta_{r}^{H}\right)+\sigma^{2}\left(\theta_{H_{a}}^{R}\right)}$$

All analysis and calculations from the equations above were conducted using the MATLAB software (Mathworks Inc., Natick, MA, USA, R2021b).

We calculated a percentage difference to examine changes between AAD (head, thoracic, lumbar, and pelvis roll) and AI values (head to thoracic, thoracic to lumbar, and lumbar to pelvis roll) in healthy individuals and survivors of stroke pre- and post-training (Equation (6)).


(6) $$\text{\% difference}=\frac{\;\text{healthy}-\text{stroke }}{(\text{healthy}+\text{stroke })/2}\times 100\text{\%}$$

### 2.3. Statistical Analysis

Participants’ data were tabulated in Microsoft Excel (Version 16.26) and then analyzed using RStudio (version 4.2.2). For both groups (i.e., healthy older adults and survivors of stroke), for each test condition, trials were pooled, from which means and standard errors were computed. Differences were determined between the assessments before (pre) and after (post) training; specifically, significant differences were observed as *p* values <0.05. Since the data were not normally distributed, a rank sum test was used. The Wilcoxon significance test (rank sum) was used to determine the significance for the healthy group pre and post for each stance condition and the stroke group pre and post for each stance condition. The Holm–Bonferroni method was used to crosscheck all significant findings to eliminate type I errors.

## 3. Results

Comparisons pre-training and post-training, for both healthy individuals and survivors of stroke, are shown in Tables 3 and 4. Tables 3 and 4 show % differences in AADs (all decreases), as well as differences in AIs (predominantly decreases), for healthy versus stroke participants. In terms of AADs (for the head, thorax, lumbar, and pelvis), the healthy older individuals had lower values compared to the survivors of stroke. This could be interpreted as an indication that the healthy older adults were better able to stabilize their body segments in space.

Table 3 shows the observable differences in AADs (decreases), and differences in AIs (predominantly decreases) for healthy versus stroke participants is shown in Table 4.

**Figure 2.**
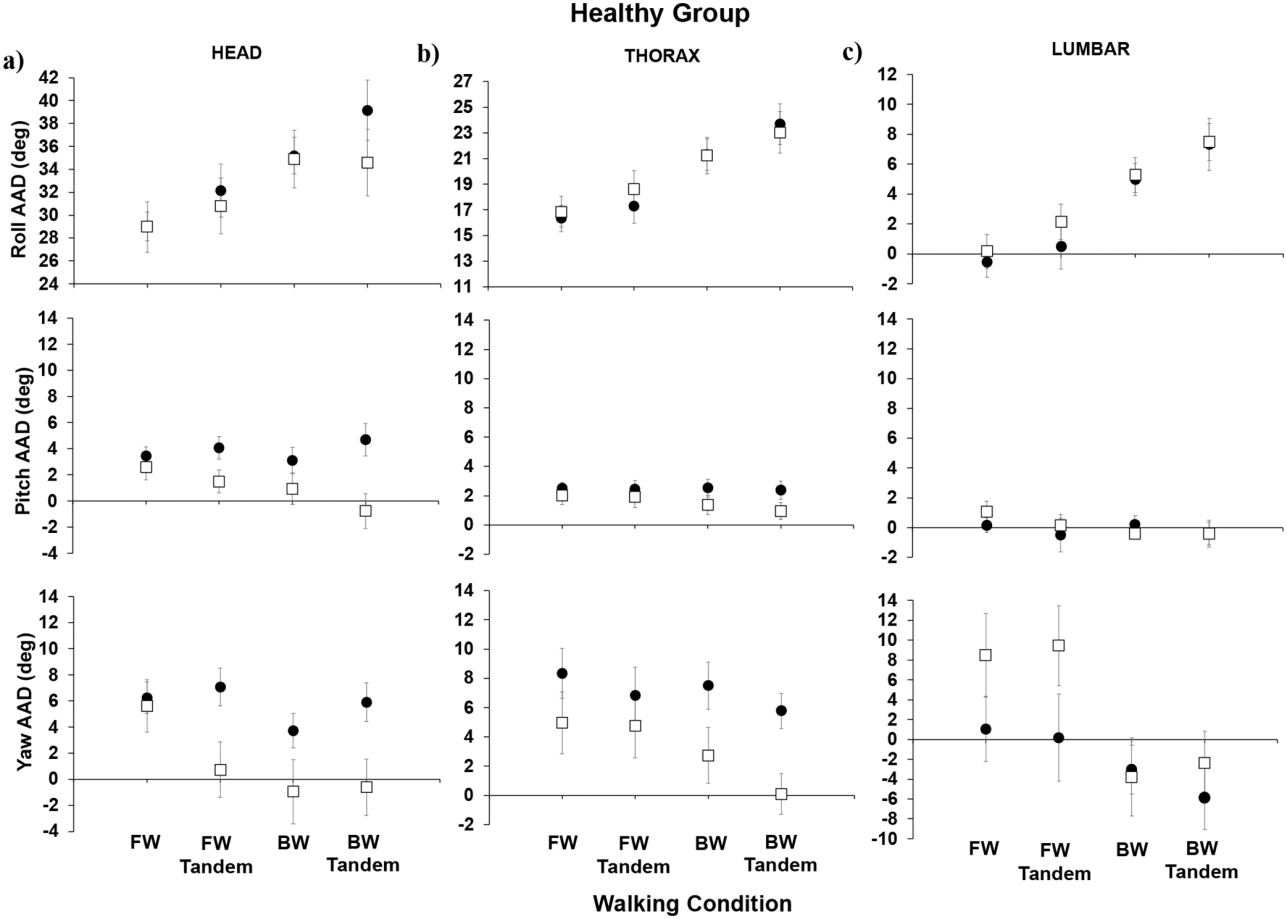
Healthy group absolute angle with deviation (AAD) across subjects for (**a**) head, (**b**) thorax, and (**c**) lumbar segments; means and standard errors are shown for pre-training (black circles) and post-training (open squares). Walking conditions listed on the x-axis are forward (FW), forward tandem (FW tandem), backward (BW), and backward tandem (BW tandem) walking.

**Figure 3.**
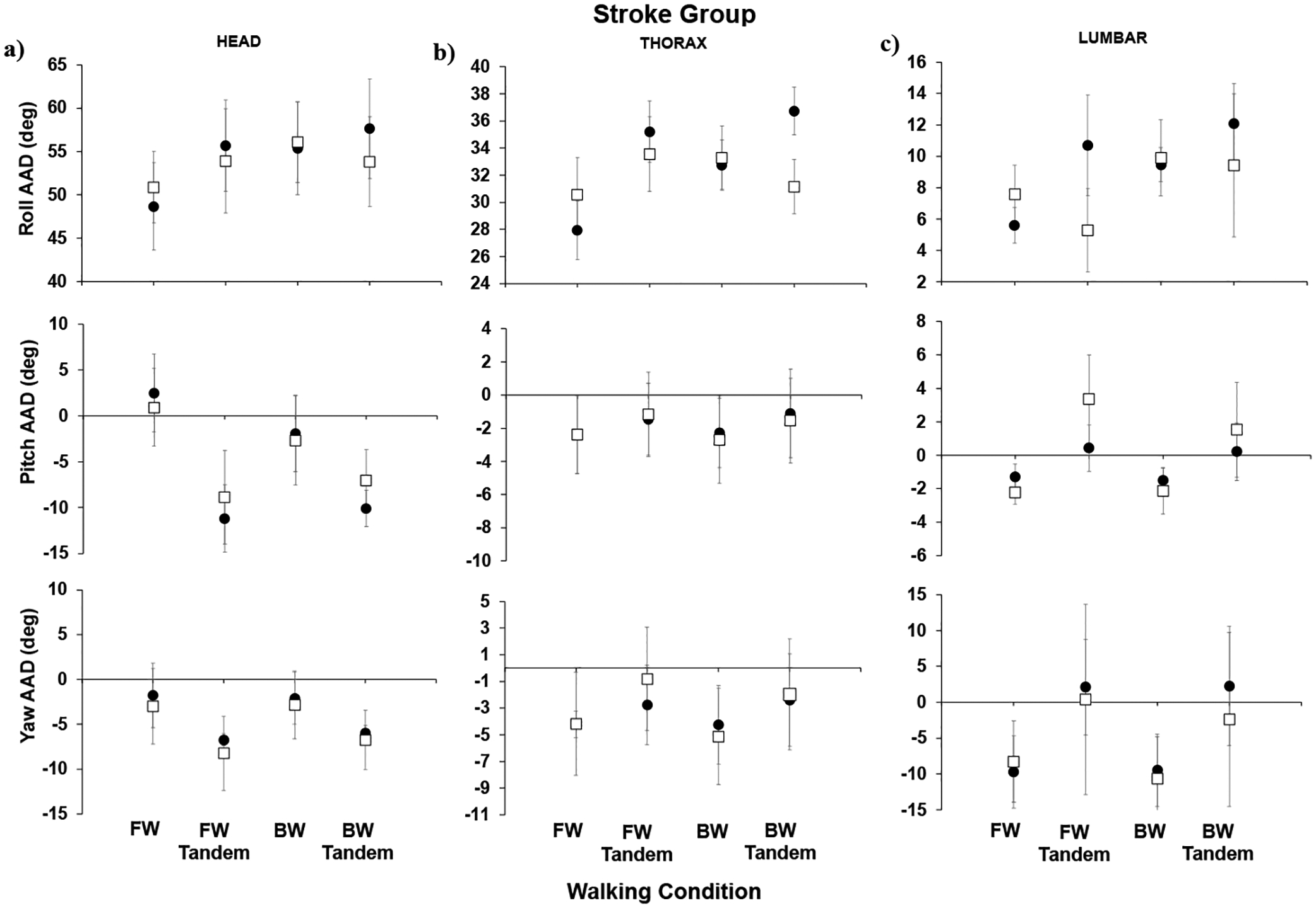
Stroke group angle with deviation (AAD) across subjects: (**a**) head, (**b**) thorax, and (**c**) lumbar segments; means and standard errors are shown for pre-training (black circles) and post-training (open squares). Walking conditions listed on the x-axis are forward (FW), forward tandem (FW tandem), backward (BW), and backward tandem (BW tandem) walking.

Below, we show the AADs for the healthy group (Figure 2) and for the stroke group (Figure 3) for forward walking (easiest), forward-tandem, backward walking, and backward-tandem (most difficult) for the head, thorax, and lumbar segments. For the healthy group results (Figure 2), when comparing post- to pre-training, significant decreases were observed in the head pitch AAD (*p* = 0.012) and head yaw AAD (*p* = 0.023) for the most difficult walking condition, backward-tandem walking. For post- compared to pre-training for the healthy individuals’ thorax, no significant difference was seen in the roll AAD or pitch AAD; however, significant decreases were seen in the thorax yaw AAD (*p* = 0.007) for backward-tandem walking. For the thorax (regular), the backward walking yaw AAD also showed a significant difference (*p* = 0.023). For the lumbar segment, both the roll AAD and pitch AAD did not show significant changes; for the lumbar segment yaw AAD, a significant increase in yaw was seen for the forward-tandem walking condition (*p* = 0.036).

**Figure 4.**
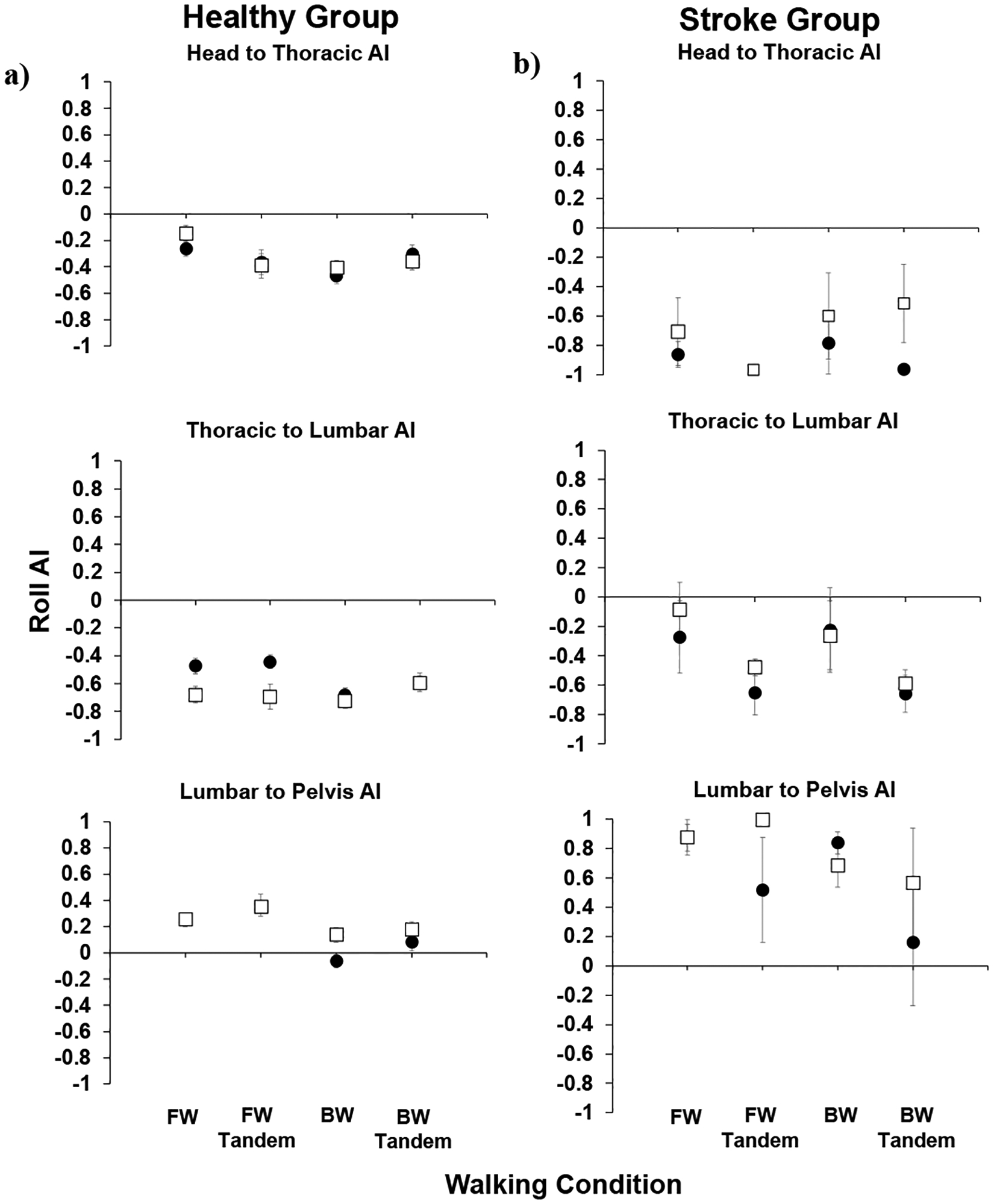
Roll anchoring indices (AI) for (**a**) healthy and (**b**) stroke groups; means and standard errors are shown for pre-training (black circles) and post-training (open squares). Walking conditions listed on the x-axis are forward (FW), forward tandem (FW tandem), backward (BW), and backward tandem (BW tandem) walking.

For the stroke group results (Figure 3), in comparing post- to pre-training AADs (roll, pitch, and yaw), no significant differences were observed. In Figure 4, the roll AIs are shown for the healthy and stroke groups. We focused on body movements in the roll plane due to its link to the fall-risk in aging individuals.

In the healthy group, for the head to thoracic roll AI, there were no significant differences observed post- versus pre-training. Further, all AIs were negative, indicating stabilization on the inferior segment as opposed to in space. For head to thoracic and thoracic to lumbar roll AIs, they were all negative in value. Further, all AIs were either positive or insignificantly different than zero, indicating better stabilization in space than on the inferior segment.

For the stroke group, all head to thoracic roll AIs were negative. For the thoracic to lumbar AI, all AIs were insignificantly different post- compared to pre-training. Further, all AIs were negative (stabilizing on the inferior segment) or insignificantly different from zero (en bloc movement). For the lumbar to pelvis, AIs were generally positive, indicating stabilization in space than on the inferior segment.

## 4. Discussion

The aim of our current study was to determine head and trunk equilibrium strategies for older individuals that were healthy or were survivors of stroke, pre- and post-sensory training. Here, we were interested in developing an understanding of the biomechanical postural control strategies used to maintain balance in aging adults, by examining whether metrics (i.e., AADs and AIs) could be used to assess aging posture. Our study’s main limitation was the smaller sample size of the stroke population. However, the results show an initial indication that, for aging individuals, training could lead to improved head and body stabilization in space post-training in the healthy population.

Our study provides an indication that metrics such as AADs and AIs can be used to assess stabilization in older adults. Further, the results show an initial indication that these measures could be used to assess the effects of aging individuals’ balance training and also their levels of impairment (e.g., healthy versus stroke). From our results, we observed that the postural strategy differed in healthy older adults compared to older adults who had suffered a stroke.

Although there had been previous studies to investigate postural control and balance in healthy persons and survivors of stroke, angular dispersion and anchoring strategies had not been explored toward characterizing postural control, nor comparisons made using these metrics described above, AADs and AIs. In our study, we observed that healthy participants had lower values of AADs (meaning less movement) for head, thoracic, lumbar, and pelvis roll both prior to and post-training. This was true for all walking conditions (i.e., forward, forward-tandem, backward, and backward-tandem). For the healthy group, significant decreases were observed in the head pitch AAD and head yaw AAD for the most difficult walking condition, backward-tandem walking, post-training. Significant decreases were also seen in the thorax yaw AAD for backward-tandem walking. In the healthy group, for the head to thoracic roll AI, there were no significant differences observed post-versus pre-training. For the stroke group, in comparing post- to pre-training AADs, no significant differences were observed. Further, for the survivors of stroke, there were no significant differences observed post- versus pre-training. However, it was observed that, overall, the healthy group had lower values of AADs than survivors of stroke both pre- and post-training. This leads us to believe that AADs may be more sensitive to observe changes in older adults. In terms of future studies, the research could be expanded to explore other aging populations—for example, persons with Parkinson’s disease—and other demographics of persons with balance and/or mobility disorders.

## Figures and Tables

**Table 1.** Demographics of participants that had suffered stroke from [22].

Subject	Age	Male/Female	Type of Stroke/Notes	Time Elapsed since Stroke (Years)	Ethnicity	Fall and/or Fall-Related Injury (within Past 5 Years)	Dizziness or Vertigo	Ailments	Activities	Vision
S1	69	Male	Aneurysm of the left internal carotid artery; Subarachnoid hemorrhage; suffered subarachnoid bleed in right hemisphere; uses ankle foot orthosis (AFO) and a cane on occasion	33	Caucasian	No	No	No	Walk in mall about 35’, PT work 1 or 2x/month	Glasses
S2	65	Male	Cerebellar stroke; uses cane, however, is able to walk without it, also regularly exercises	3	Caucasian	No	Yes	No	Daily walking	No
S3	61	Male	Weakness on left side due to stroke; uses stimulation as opposed to AFO, also very active with regular exercise	3	Caucasian	Yes	No	Weakness on left side	Personal trainer, pilates	Glasses
S4	56	Male	Left thalamic intraparenchymal hemorrhage in; multiple lacunar infarcts, microhemorrhages, and small vessel disease; no cane nor walker	1,3	African American	No	Yes	No	Yes	Glasses
S5	81	Female	Suffered a small acute stroke in the high right frontal lobe with no hemorrhage; may have suffered a second stroke, but did not stay for diagnostic; no cane nor walker used	0.75	Caucasian	Yes	Periodic	Feet pronate	Water Aerobics	Glasses
S6	72	Male	Suffered a stroke but not provide doctor’s assessment	10	Caucasian	Yes	Did not provide	Did not provide	Did not provide	Glasses
S7	59	Female	Suffered a stroke but did not provide additional information on type of stroke	26	Caucasian	No	No	hearing loss/vision	OT/PT, treadmill, bike	Glasses

**Table 2.** Demographics of healthy participants from [22].

Subject	Age	Male/Female	Ethnicity	Fall and/or Fall-Related Injury (within Past 5 Years)	Dizziness or Vertigo	Ailments	Activities	Vision
H1	78	Female	Caucasian	Yes	No	Poor dorsaflexion in left foot	Water aerobics and walking	Glasses
H2	65	Female	Caucasian	No	No	No	No	Glasses
H3	70	Female	Caucasian	No	No	Did not provide	Did not provide	No
H4	70	Female	Caucasian	No	No	Unsure	Visits to Wellness Center and Jazzercise	Glasses
H5	67	Female	Caucasian	No	No	No	Weight lifting, yoga, and hiking	Glasses (for reading only)
H6	67	Female	Caucasian	No	No	No	Walking and yoga	Glasses
H7	71	Female	Caucasian	Yes	Yes	Hearing loss	Weight bearing exercises and walking	No
H8	70	Male	Caucasian	Did not provide	Yes	Chronic disk impairment	1x/week with med ex trainer	Glasses (for reading only)
H9	66	Female	Caucasian	No	No	Uneven leg strength	Working out with trainer, walking, and yoga	Glasses (for reading only)
H10	72	Female	Caucasian	No	No	No	Yes	Glasses
H11	63	Female	Caucasian	No	No	No	Walking, gardening, yoga	No
H12	68	Female	African American	No	No	Arthritis	No	Glasses
H13	63	Female	African American	No	No	No	Yes	Glasses (for reading only)
H14	74	Female	Caucasian	No	No	Arthritis	Water aerobics	Glasses
H15	80	Female	Caucasian	No	No	No	Water aerobics	Glasses
H16	63	Female	Caucasian	No	No	No	Water aerobics	No
H17	78	Female	Caucasian	No	No	No	No	Glasses
H18	71	Female	European	No	Periodic	No	Walking, jogging, and biking	No
H19	71	Female	Caucasian	No	No	No	Low-impact aerobics and yoga	Glasses
H20	64	Female	African American	No	No	No	Balance and strength exercises	Glasses
H21	62	Male	African American	No	No	No	1–2x/week of exercise	Blind in one eye
H22	83	Female	Caucasian	Yes	No	No	Water aerobics	No
H23	68	Male	Caucasian	No	No	Arthritis	Stretching, stationary bike, and yoga	Glasses
H24	65	Female	African American	Did not provide	Did not provide	Did not provide	Did not provide	Did not provide
H25	67	Female	Caucasian	No	No	No	Strength and flexibility, cycling, and treadmill walking	No
H26	68	Male	African	Did not provide	Did not provide	Did not provide	Did not provide	Glasses
H27	75	Male	Caucasian	Yes	No	No	Jogging and swimming	Glasses

**Table 3.** Percent difference comparison of healthy versus stroke mean AAD results (head, thoracic, lumbar, and pelvis) and healthy versus stroke pre- and post-training.

% Difference								
	Head Roll AAD		Thoracic Roll AAD		Lumbar Roll AAD		Pelvis Roll AAD	
	Pre	Post	Pre	Post	Pre	Post	Pre	Post
Forward	−50.6	−54.9	−52.4	−57.8	−245.9	−191.4	−17.1	−15.6
Forward Tandem	−40.9	−49.2	−47.0	−48.6	−168.9	−112.1	−29.0	−13.9
Backward	−44.6	−46.5	−42.2	−44.3	−62.1	−61.1	−19.8	−17.2
Backward Tandem	−38.3	−43.5	−43.2	−29.9	−49.2	−23.1	−33.9	−11.9

**Table 4.** Percent difference comparison of healthy versus stroke mean AI results (head to thoracic, thoracic to lumbar, and lumbar to pelvis) results pre- and post-training.

% Difference						
	Head to Thoracic Roll AI		Thoracic to Lumbar Roll AI		Lumbar to Pelvis Roll AI	
	Pre	Post	Pre	Post	Pre	Post
Forward	−106.4	−130.7	53.9	156.0	−111.6	−109.2
Forward Tandem	−89.7	−84.7	−38.1	36.8	−34.3	−95.2
Backward	−50.5	−38.9	100.7	94.3	−233.6	−132.6
Backward Tandem	−103.6	−36.2	−11.1	0.8	−64.8	−105.2

## Data Availability

The data presented in this study are available on request from the corresponding author.
